# Rapid Eating Assessment for Participants [shortened version] scores are associated with Healthy Eating Index-2010 scores and other indices of diet quality in healthy adult omnivores and vegetarians

**DOI:** 10.1186/s12937-018-0399-x

**Published:** 2018-09-28

**Authors:** Carol S Johnston, Courtney Bliss, Jessica R Knurick, Cameron Scholtz

**Affiliations:** 1 Nutrition Program, College of Health Solutions, Arizona State University, 500 N. 3rd Street, Phoenix, AZ 85004 USA; 2 Feeding Bliss ℅ Thrive Therapy, 3420 E. Shea Blvd. Unit 188, Phoenix, AZ 85028 USA; 3 Toolbox Genomics, 75 Broadway Street #251, San Francisco, CA 94115 USA

**Keywords:** Diet quality, Rapid eating assessment for participants, Healthy eating index, Vegetarian

## Abstract

The Healthy Eating Index-2010 is a measure of diet quality as portrayed by the Dietary Guidelines for Americans; however, computing the Healthy Eating Index score is time consuming and requires trained personnel. The Rapid Eating Assessment for Participants [shortened version] is a simple measure that quickly, in less than 10 min, assesses diet quality in a clinical or research setting. This research evaluated the degree of correlation between these two methods of scoring diet quality, as well as between these methods and other indicators of diet quality, including the nutrient density of the diet, the dietary potential renal acid load, urine pH, and plasma vitamin C concentrations. The research design was a secondary data analysis, and participants were healthy adults (*n* = 81) self-classified as omnivorous, vegetarian, or vegan. Confounding variables were identified and controlled using partial correlations. The two methods of scoring diet quality were significantly correlated (*r* = 0.227, *p* = 0.047). Both the Healthy Eating Index and the Rapid Eating Assessment for Participants scoring methods were correlated to nutrient density of the diets (*r* = 0.474 and *r* = 0.472 respectively, *p* < 0.001) as well as to the dietary potential renal acid load and urinary pH (r ranging from 0.304–0.341, *p* ≤ 0.002). The Rapid Eating Assessment for Participants, but not the Healthy Eating Index, was significantly correlated to plasma vitamin C concentrations (*r* = 0.500, *p* < 0.001 and 0.192, *p* = 0.095 respectively). These results in combination with ease of use and low cost suggest that the Rapid Eating Assessment for Participants measure is a useful tool for assessing diet quality.

## Background

Healthcare providers and researchers would benefit from an easily administered and meaningful tool to assess diet quality. The Healthy Eating Index (HEI was developed in 1995 and is a premier tool for scoring diet quality. The HEI scores diet quality on a 100-point scale with full points given for a diet that closely follows the Dietary Guidelines for Americans (DGA) [1]. The HEI was updated with the release of the 2010 DGA and termed HEI-2010 [2, 3], and the HEI-2015 version, to correspond with the 2015–2020 Dietary Guidelines was recently released [4] However, this measure is time consuming to administer and requires trained personnel to calculate scores [1, 2]. Moreover, since there is no standardization for the number of items and score range, there is concern that HEI scores may not be comparable between versions or between trials [5].

The Rapid Eating and Activity Assessment for Patients (REAP) survey was developed in 2003 to quickly assess nutrient intake and assist in brief lifestyle counseling by a healthcare provider [6]. The survey contained 27 questions scored from 1 to 3 to assess intake of whole grains, calcium-rich foods, fruits and vegetables, fat, saturated fat and cholesterol, sugary beverages and foods, sodium, and alcoholic beverages. The summed scores for the 27 questions estimated diet quality with higher scores indicating higher diet quality (score range, 27–81). This measure correlated well with the original HEI measure (*r* = 0.490; *p* = 0.0007), and test-retest reliability for respondents following a 1–3 week interval was strong, *r* = 0.860 [7]. In 2004, the REAP was shortened to 13 scored questions by deleting questions on type of dairy, type of ground beef, removing skin and fat on poultry and meats, fat-free substitutes, alcoholic drinks, and physical activity [8]. The intent of this revision was to focus on food intake and the practicality of use in low-literacy populations. Total scores range from 13 to 39 for the shortened REAP questionnaire, renamed Rapid Eating Assessment for Participants – shortened version (REAP-S), and it was validated against nutrient intakes using the 1998 Block food frequency questionnaire in medical students (*n* = 110) [8].

The purpose of this study was to examine the relationship between the REAP-S and the HEI-2010 for scoring diet quality in healthy adults across a range of dietary patterns. Diet quality scores for both measures were also related to other indicators of diet quality: nutrient density of the diet [9], the Potential Renal Acid Load (PRAL) [10], urine pH [11], and plasma vitamin C concentrations [12, 13]. In addition, the REAP-S and the HEI-2010 were related to common health biomarkers such as body mass index (BMI), blood pressure, fasting plasma glucose and triglyceride concentrations and to nutrient intakes. Healthcare providers and researchers would benefit from a tool that effectively measures diet quality quickly and with ease.

## Methods

### Participants and study design

This secondary analysis was conducted using data from a cross-sectional study that examined correlates of bone mineral density in healthy vegetarian and omnivorous adults [14]. This data set represented a broad range of dietary plans providing a wide range of diet quality for this comparison. Recruitment took place at a large university in Phoenix, Arizona and the surrounding community in 2013. Volunteers were recruited using emails, flyers and online forums for local vegetarian and vegans. Individuals interested in participating were directed to complete a brief online questionnaire. Healthy, non-smoking adults between the ages of 18 and 50 years who had been following their current diet (omnivore, vegetarian or vegan) for at least six months were invited to the study site to learn more about the study. Volunteers were excluded if they had a BMI greater than 30 kg/m^2^, were pregnant or lactating within the six months prior to screening, had irregular menstrual cycles, were athletes, or were taking prescription medications (e.g. insulin, corticosteroids, sulfonylureas, sodium valproate, thyroid replacement therapy, beta-blockers, ACE inhibitors, diphenhydramine, lithium carbonate, thiazolidinediones, and/or cyproheptadine). The study was approved by the Institutional Review Board at Arizona State University, and all participants provided written consent prior to data collection.

The data reported herein were collected at two site visits that occurred within one week. At the first visit, trained personnel completed the anthropometric measurements using standardized procedures, and participants completed questionnaires and a 24-h recall. At the second visit, participants presented at the testing facility in a fasted state (no food or beverage with the exception of water for 10 h), and a venous blood sample was collected by the study nurse. Participants also provided a 24-h urine sample at the second visit (all urine voids from the second void on the day prior to the second visit through the first urine void of the day of the second visit). A total of 83 participants were enrolled in the study; however, one participant did not complete the REAP-S questionnaire, and a second participant did not provide a 24-h dietary recall; hence, the total sample size used in this secondary data analysis was 81 (27 omnivores, 26 vegetarians and 28 vegans).

### Anthropometric data, blood pressure, blood and urine analysis

With participants wearing light clothing and barefoot, height was measured using a wall-mounted stadiometer, and body weight was measured using a calibrated scale (model TBF-300A, Tanita Corporation, Tokyo, Japan). Waist measurements were taken using a flexible tension tape at the minimal circumference [15]. Following a 10-min seated rest, blood pressure was recorded for three consecutive measurements with the 2nd and 3rd measurements averaged for the recorded values (Automatic Digital Blood Pressure monitor, Medline Industries Inc., Mundelein, IL). Plasma vitamin C was measured using the 2,4-dinitrophenylhydrazine colorimetric method [16]. Fresh plasma was deproteinized by mixing with an equal aliquot of refrigerated 10% trichloroacetic acid followed by centrifugation at 3500 g and 0 °C for 20 min. The resultant supernatant was stored at − 80 °C until analysis. Fasting blood samples were also analyzed for total cholesterol, high-density lipoprotein cholesterol, low-density lipoprotein cholesterol, triglycerides, and serum glucose using a point-of-care COBAS C111 chemistry random access autoanalyzer (Roche Diagnostics, Indianapolis, IN). The WTW Chekmite pH -20 Sensor pH meter was used to determine urine pH (Nova pH, Woburn, MA, USA).

### Diet analysis

The REAP-S questionnaire was completed for the previous week’s intake and scored by summing responses for the first 13 questions. Responses of ‘usually/often’ received 1 point, ‘sometimes’ received 2 points, and ‘rarely/never or does not apply to me’ received 3 points [8]. Possible scores ranged from 13 to 39 with a higher score indicating a higher diet quality.

The unannounced 24-h dietary recall was conducted by a trained nutrition professional. Food and beverage intake was systematically recalled, and participants were asked for detailed information on food sources and brands, serving size, preparation method, and condiment use. Diet data were entered into The Food Processor® Nutrition and Fitness Software by ESHA Research, Inc. (version 10.11, ©2012) for scoring of the HEI-2010. Food Processor automatically calculates servings by food groups via MyPlate. The investigator used the auto-populated MyPlate servings as the basis but then spot-checked each food against the MyPlate website. Any foods that were not in the Food Processor database were looked up online and manually added to the 24-h dietary record. If the nutrition facts for the foods were not easily found through an Internet search, the investigator looked up three similar products and took the average of the three measures as the surrogate. To minimize investigator bias, standard defaults were used for dietary items not in the database or not clearly reported.

Diet quality was scored using the HEI-2010, a rubric for diet quality as indicated by the 2010 DGA [2, 17]. The HEI-2010 is composed of one hundred possible points across twelve categories divided into adequacy components (*n* = 9) and moderation components (*n* = 3). Scores for each component were calculated on a sliding scale based on the adherence to the DGA recommendations. The nine adequacy components were total fruit, whole fruit, total vegetables, greens and beans, whole grains, dairy, total protein foods, seafood and plant proteins, and fatty acids. For the adequacy components (with the exception of fatty acids), zero consumption of the food group in question yielded zero points, consumption at 100% of the standard per thousand kilocalories yielded full points and any intake between these received an incremental score [2]. Scores for each of the adequacy components ranged from 5 to 10. For fatty acids (the only nutrient among the adequacy components), the 15th percentile of the target population 1-day intake was used as the zero reference [2]. The three categories of the moderation components were refined grains, sodium and empty calories. Scores for the moderation components ranged from 10 to 20 points and were scored inversely of the adequacy groups; intakes at the level of the standard or lower received the maximum points while higher intakes received proportionately lower scores [2].

The nutrient density of the diet was quantified using the Nutrient Rich Food Index that is based on nine nutrients to ‘encourage’ and three nutrients to ‘limit’ (NRF9.3): (Protg/50 + Fibg/25 + VitARAE/1515 + VitCmg/60 + VitEaTocomg/20 + Calcmg/1000 + Ironmg/18 + Magnmg/400 + Potmg/3500 – SatFatg/20 – Sugarg/50 - Sodmg/2400) * 100 [9]. To adjust for energy intake, the index was divided by kcal/100. PRAL was calculated from dietary intake using the equation: (0.49 protein (g)) + (0.037 P (mg)) − (0.021 K (mg)) − (0.026∙Mg (mg)) − (0.013 Ca (mg)) [18].

### Statistical analyses

Date are reported as the mean ± SD. Data normality was evaluated by inspection of Q-Q plots and histograms. Confounding variables, identified by correlational analyses, were noted for the HEI index only: energy intake, body weight, and gender. Pearson or Spearman correlation coefficient analyses were used to examine the strength of correlations between diet quality measures as appropriate based on normality of the data. Partial correlation analyses were used to control for confounding variables. Differences between groups were assessed using oneway analysis of variance controlling for confounding variables. If data were not normally distributed, the Kruskal-Wallis test was used. Nominal data were assessed using the Chi-square statistic. SPSS for WINDOWS (version 23; SPSS Inc., Chicago, IL, USA) was used for statistical analyses, and significance was set at *p* < 0.05.

## Results

The study sample (*n* = 81) reported adherence to one of three dietary plans: omnivore (consumed animal products daily, *n* = 27), vegetarian (excluded all flesh foods, *n* = 26), or vegan (excluded all animal foods, *n* = 28). Participants were young adults (30.9 ± 8.5 y; 70% female) of healthy weight (22.8 ± 2.8 kg/m^2^). Anthropometric measures (body weight, BMI, waist circumference, and body fat percentage) did not differ by diet group (Table 1); however, the omnivore participants were significantly younger than vegan participants (27.2 ± 6.7 and 33.9 ± 8.6 y, respectively). Age did not correlate to the diet quality measures with the exception of PRAL (− 0.324, *p* = 0.003); however, controlling for age did not erase the significant differences for PRAL between diet groups. HEI-2010 was related to several participant characteristics (body weight and gender) as well as to energy intake, and these variables were adjusted in all HEI-2010 diet quality analyses. Blood pressure and fasting glucose and triglyceride concentrations did not vary by diet group (Table 1). The only significant correlation between a biomarker and diet quality score was that for REAP-S and fasting plasma glucose (*r* = − 0.228, *p* = 0.040; Table 1). Blood cholesterol concentrations did not differ by diet group or relate to the diet quality scores (data not shown).

**Table 1:** Correlation of participant characteristics to REAP-S and HEI-2010^a^

Measure	Total (81)	Omnivores (27)	Vegetarian (26)	Vegan (28)	*P*	Coefficient, *r*	
						REAP-S	HEI-2010
Gender (M/F)	24/57	8/19	6/20	10/18	0.597	–	–
Age (y)	30.9 ± 8.5	27.2 ± 6.7^a^	31.6 ± 9.0^ab^	33.9 ± 8.6^b^	0.012	−0.064	0.025
Weight (kg)	65.0 ± 11.3	66.8 ± 12.0	63.4 ± 9.9	64.6 ± 12.0	0.542	−0.111	− 0.248*
BMI (kg/m^2^)	22.8 ± 2.8	23.5 ± 3.1	22.5 ± 2.7	22.3 ± 2.6	0.276	−0.127	−0.090
Body fat (%)	29.8 ± 7.9	29.1 ± 8.8	31.7 ± 7.7	28.8 ± 7.3	0.345	−0.174	−0.232*
Waist (cm)	80.3 ± 9.9	80.5 ± 10.6	80.2 ± 9.8	80.1 ± 9.6	0.991	−0.138	−0.124
Systolic BP (mmHg)	115.2 ± 9.6	116.6 ± 8.9	114.6 ± 10.7	114.4 ± 9.4	0.659	−0.012	−0.144
Diastolic BP (mmHg)	70.9 ± 7.4	69.7 ± 7.3	70.7 ± 6.8	72.3 ± 8.0	0.419	0.081	0.022
Plasma glucose (mg/dL)	85.5 ± 7.0	86.0 ± 7.2	85.5 ± 6.9	85.0 ± 7.1	0.864	−0.228*	− 0.082
Plasma triglycerides (mg/dL)^b^	79.0 ± 50.7	67.3 ± 31.1	77.6 ± 50.1	91.5 ± 63.6	0.258	0.050	−0.002

^a^*P* represents Oneway analysis of variance test; means with different superscripts differ significantly (LSD test, *p* < 0.05). *r* represents Pearson correlation; asterisk indicates significant correlation (*p* < 0.05). HEI-2010 analyses adjusted for energy intake, body weight, and gender

^b^Distribution not normal; nonparametric tests uses for statistical analyses (Kruskal Wallis Test and Spearman correlation)

HEI-2010 and REAP-S were significantly correlated for the study sample (*r* = 0.227, *p* = 0.047). Additionally, both HEI-2010 and REAP-S were significantly correlated to other indicators of diet quality: NRF9.3, PRAL, and urine pH (Table 2). However, only REAP-S was correlated to plasma vitamin C concentration, an additional indicator of diet quality (Table 2). Overall, REAP-S appeared to correlate more strongly with the other indicators of diet quality compared to HEI-2010 (e.g., PRAL, urine pH, plasma vitamin C, and nutrient density of the diet; absolute average correlation coefficients: *r* = 0.406 and *r* = 0.321 for REAP-S and HEI-2010 respectively). Diet quality measures differed by diet group with the exception of HEI-2010, and differences were mainly between the omnivore and vegan groups (Table 2).

**Table 2:** Diet quality indicators by diet group and their relationship to REAP-S and HEI-2010^a^

Measure	Total (81)	Omnivores (27)	Vegetarian (26)	Vegan (28)	*P*	Coefficient, *r*	
						REAP-S	HEI-2010
REAP-S	33.6 ± 3.1	31.8 ± 3.1^a^	32.7 ± 2.3^a^	36.1 ± 2.0^b^	< 0.001	–	0.227*
HEI-2010	47.4 ± 14.1	44.8 ± 13.0	47.7 ± 13.4	49.8 ± 15.8	0.458	0.227*	–
NRF9.3w	31.0 ± 25.1	23.2 ± 23.2^a^	26.0 ± 20.4^a^	44.9 ± 26.5^b^	0.002	0.474*	0.472*
PRAL	0.8 ± 33.7	19.6 ± 24.3^a^	− 1.5 ± 23.9^b^	−15.2 ± 40.5^b^	< 0.001	− 0.309*	− 0.304*
Urine pH	6.5 ± 0.5	6.2 ± 0.4^a^	6.5 ± 0.4^b^	6.7 ± 0.4^b^	< 0.001	0.341*	0.317*
Vitamin C, mg/dL	0.591 ± 0.156	0.524 ± 0.163^a^	0.592 ± 0.143^ab^	0.654 ± 0.138^b^	0.007	0.500*	0.192

^a^*P* represents Oneway analysis of variance test; means with different superscripts differ significantly (LSD test, *p* < 0.05). r represents Pearson correlation; asterisk indicates significant correlation (*p* < 0.05). HEI-2010 analyses adjusted for energy intake, body weight, and gender. Overall, the absolute average level of correlation for diet quality indices and REAP-S is higher than for diet quality indices and HEI-2010 (0.406 and 0.321 respectively)

Although total energy intake did not differ by diet plan, differences between groups were noted for macronutrient intake. Carbohydrate intake was 40% higher and protein intake was 29% lower for the vegan vs. omnivore participants (Table 3). Simple sugar intake did not differ by diet plan; yet, saturated fat intake was 63% lower and fiber was 99% higher for the vegan vs. omnivore participants. Cholesterol intakes were lower for the vegetarians in comparison to the omnivores. Many of the vitamin and mineral intake differed by diet group; exceptions included vitamin A, vitamin D, zinc, calcium, sodium, and potassium (Table 3). REAP-S correlated in a healthful direction with intakes for ten nutrients (fat, saturated fat, fiber, cholesterol, vitamin C, folate, vitamin A, vitamin E, iron, and potassium). HEI-2010 correlated favorably with four of the nutrients (fiber, vitamin C, vitamin A, and potassium).

**Table 3:** Energy and nutrient intake by diet group and their relationship to REAP-S and HEI-2010^a^

Measure	All (81)	Omnivores (27)	Vegetarian (26)	Vegan (28)	*P*	Coefficient, *r*	
						REAP-S	HEI-2010
Energy, kcal	2073 ± 647	2108 ± 727	2042 ± 558	2069 ± 665	0.991	−0.153	−0.434*
Protein, g	77.9 ± 37.1	96.8 ± 47.5^a^	68.4 ± 23.9^b^	68.6 ± 29.0^b^	0.015	−0.195	0.087
Carbohydrate, g	287.7 ± 109.3	239.1 ± 97.5^a^	286.6 ± 87.1^ab^	335.6 ± 120.2^b^	0.007	0.139	−0.081
Sugar, g	92.6 ± 48.1	81.7 ± 42.9	90.0 ± 37.9	105.4 ± 58.7	0.221	−0.101	0.045
Fat, g	71.0 ± 36.5	86.3 ± 42.8^a^	70.8 ± 29.2^ab^	56.4 ± 30.6^b^	0.016	−0.411*	0.001
Saturated fat, g	19.8 ± 14.8	30.0 ± 16.7^a^	18.4 ± 10.1^b^	11.2 ± 10.3^c^	< 0.001	−0.567*	− 0.211
Fiber, g	36.4 ± 19.8	24.8 ± 11.8^a^	34.6 ± 14.6^b^	49.4 ± 22.7^c^	< 0.001	0.437*	0.441*
Cholesterol, mg	138.4 ± 215.3	330.8 ± 263.6^a^	87.7 ± 109.2^b^	0 ± 0^c^	< 0.001	− 0.631*	− 0.190
Vitamin C, mg	172.6 ± 151.8	149.2 ± 134.0^a^	140.4 ± 128.2^a^	225.2 ± 177.0^b^	0.049	0.237*	0.275*
Folate, DFE	412.2 ± 390.9	290.2 ± 281.0^a^	415.8 ± 420.7^ab^	522.1 ± 428.0^b^	0.052	0.381*	0.129
Vitamin B12, μg	3.5 ± 5.8	4.9 ± 8.0^a^	2.3 ± 3.4^b^	3.3 ± 5.1^b^	0.051	−0.119	−0.125
Vitamin A, RAE	752.4 ± 875.6	518.9 ± 486.6	709.0 ± 788.1	1017.8 ± 1157.1	0.337	0.379*	0.422*
Vitamin E, mg	11.6 ± 14.5	8.3 ± 11.9^a^	8.8 ± 7.8^a^	17.3 ± 19.4^b^	0.008	0.241*	0.058
Vitamin D, μg	1.7 ± 2.6	2.2 ± 2.9	1.3 ± 2.5	1.7 ± 2.3	0.089	0.023	0.115
Iron, mg	19.1 ± 11.4	15.0 ± 7.8^a^	18.5 ± 9.5^ab^	23.6 ± 14.2^b^	0.023	0.241*	0.170
Zinc, mg	7.6 ± 6.4	8.7 ± 8.2	5.6 ± 3.8	8.5 ± 6.1	0.217	0.163	0.181
Calcium, mg	824.8 ± 449.7	938.6 ± 516.0	746.3 ± 421.7	787.9 ± 397.3	0.204	0.030	0.029
Sodium, mg	3040.9 ± 1589.8	3742.7 ± 1877.3	2870.7 ± 1389.3	2522.2 ± 1228.1	0.058	−0.063	−0.158
Potassium, mg	2361.9 ± 1517.2	2047.6 ± 1152.7	2135.0 ± 1219.7	2875.5 ± 1934.4	0.238	0.292*	0.395*

^a^Differences between means assessed by Kruskal Wallis Test; means with different superscripts differ significantly (Mann-Whitney U test; *p* < 0.05). *r* is for Spearman correlation. Significant correlations are asterisked (*p* < 0.05). HEI-2010 analyses were adjusted for energy intake, body weight, and gender

## Discussion

These data demonstrated that REAP-S and HEI-2010 scores were significantly correlated across a range of diet patterns. An earlier study also reported a significant correlation between the original, long-version REAP with the original HEI [7]. Knowing that these measures of diet quality are correlated is useful since REAP-S is quick, less costly, and simple to administer and score in comparison to the HEI-2010. Researchers can utilize REAP-S to quantify diet quality in a study population and track change in diet quality over time in intervention or cohort trials. In addition, health professionals can utilize REAP-S in their practice to quickly assess diet quality, identify diet concerns, and communicate diet strategies with their patients. Conversely, the HEI scoring process can be time-consuming and involves coding procedures that can vary between investigations [5].

The Center for Nutrition Policy and Promotion, a program of the U.S. Department of Agriculture, created the HEI index to monitor the diet quality of the U.S. population. In this context, diet quality was defined as conformance to the DGA. The HEI was updated three times since its inception in 1995 to reflect changes to the DGA in 2005, 2010, and 2015. Based on data from the National Health and Nutrition Examination Survey, HEI mean scores for American adults ranged from 56 in 2006 to 59 in 2014 [19]. In the present report, mean HEI-2010 was 47.4 ± 14.1 and ranged from 44.8 ± 13.0 to 47.7 ± 13.4 and 49.8 ± 15.8 for the omnivores, vegetarians, and vegans respectively. Kim et al. recently reported a similar mean HEI-2010 score for college students in the Southern U.S. (*n* = 110, HEI average, 44) [20]. Mean HEI-2010 scores for adults from Baltimore neighborhoods (*n* = 1358) ranged from 45 to 48 based on gender and ethnicity [21]. In a study of Belgium adults, mean HEI-2010 scores were 46 and 54 for age and gender-matched omnivores (*n* = 69) and vegetarians (*n* = 69) [22].

The REAP measure was developed through an initiative sponsored by the Nutrition Academic Award Program to improve nutrition training in U.S. medical schools [6]. Although there are reports describing the development and validation of the REAP-S measure [7, 8, 23], there are no investigations using REAP-S to score diet quality among U.S. adults. The REAP-S scores presented herein suggest that the mean REAP-S score for adults consuming a typical omnivorous diet is 32, which serves as a base of comparison for future trials. The correlation coefficient between REAP-S and HEI 2010 reported herein is lower than that reported for the original versions of these measures: 0.23 and 0.49 respectively. However, the latter comparison utilized a 3-day diet record for dietary assessment, which may yield more comprehensive diet data [24].

The fact that REAP-S scores correlated to measures of diet quality aside from HEI-2010 provides additional support for the use of REAP-S to assess diet quality. The use of nutrient density profiling to assess diet quality represents a more global view regarding diet quality when compared to the DGA and the HEI-2010. For this measure, nutrient-rich diets are defined generally as those that contain more nutrients than calories and are low in fat, sugar and salt [25]. The NRF9.3 index was used in the present trial to profile the nutrient density of the participants’ diets [9]. Both REAP-S and HEI-2010 were significantly correlated to this index when adjusted for dietary energy (*r* = 0.474 and 0.472 respectively). The dietary acid load is another marker of diet quality since it is strongly related to fruit and vegetable intake [26, 27]. In this trial, both a diet index, PRAL, and urine pH were used as surrogates for dietary acid load [11, 18]. Again, both REAP-S and HEI-2010 were significantly correlated (inversely) to the acid load. Lastly, plasma vitamin C is considered an objective measure for diet quality since it is found only in dietary fruits and vegetables [12, 13]. In the present trial, REAP-S, but not HEI-2010, was significantly correlated to plasma vitamin C concentrations (*r* = 0.500 and 0.192 respectively).

Although REAP-S differentiated between dietary patterns, HEI-2010 did not possess this discriminant ability. However, others have reported significant differences in HEI-2010 scores between omnivores and vegetarians in large population samples [28, 29]. In these investigations, total HEI-2010 scores were 11 to 16 points higher among vegetarian populations in comparison to meat-eaters whereas in the present study there was a maximum 5 point difference in mean scores between diet groups. In addition to the small sample size limitation of the present study, the sample represented young, healthy, slender adults; hence, these results may not extend to older adults, overweight/obese adults, or patient populations. Additional research is needed to appraise the strength and validity of the REAP-S in more diverse participant populations and for use in randomized clinical trials.

With the release of the 2015–2020 DGA, the HEI-2010 index was recently updated to include scoring for added sugars and saturated fats as emphasized in the new guidelines [4]. The HEI-2015 became available in 2017; however, the data reported herein were collected in 2013 and this secondary analysis was initiated in 2014. Although a premier index for diet quality, the HEI is a tool to monitor compliance with the DGA; as such, the HEI continuously evolves over time. The present report examined the relationship between the REAP-S and the HEI-2010 for scoring diet quality in healthy adults across a range of dietary patterns and was not focused on the degree of adherence to the DGA. The data indicate that REAP-S appears to correlate to various measures of diet quality, including the HEI-2010.

A study strength and distinction was the comparison of REAP-S and HEI-2010 to other indices of diet quality. Absolute correlation coefficients ranged from 0.309 to 0.500 for REAP-S and the four other diet quality measures (*p* ≤ 0.002). These data provide evidence of the concurrent criterion validity of REAP-S. For HEI-2010, the correlation coefficients with the other four measures of diet quality were generally lower (ranging from 0.192 to 0.472), and one of these comparisons did not attain significance (i.e., plasma vitamin C concentrations were not correlated to HEI-2010 scores). Further analyses demonstrated that intakes of ten of the 18 nutrients assessed in this study favorably correlated with REAP-S; yet, of the three nutrients targeted by the DGA (sugar, saturated fat, and sodium), only saturated fat intake correlated significantly with REAP-S (*r* = − 0.567). In comparison, intakes for four of 18 nutrients (i.e., fiber, vitamin C, Vitamin A, and potassium) correlated significantly to HEI-2010 but sugar, saturated fat or sodium were not among these nutrients. It should be noted that previous investigations reported modest correlation coefficients for HEI scores and saturated fat intake (*r* = 0.24) [30] and for HEI scores and plasma vitamin C (*r* = 0.21) [13].

## Conclusions

This research demonstrated that REAP-S scores correlate with HEI-2010 scores in a healthy adult population consuming both plant and animal-based diets. Furthermore, unlike the HEI-2010 measure, the REAP-S measure discriminated between omnivorous and vegan diets. REAP-S also correlated favorably with four other indicators of diet quality (PRAL, urine pH, plasma vitamin C, and nutrient density of the diet) as well as with the intake of various nutrients including saturated fat. These results in combination with ease of use and low cost suggest that the REAP-S measure is a useful tool for rapid assessment of diet quality.

## Abbreviations

BMI: Body mass index
HEI: Healthy eating index
NRF: Nutrient rich food index
PRAL: Dietary potential renal acid load
REAP-S: Rapid eating and activity assessment for patients short version survey
